# Sc3.0: revamping and minimizing the yeast genome

**DOI:** 10.1186/s13059-020-02130-z

**Published:** 2020-08-13

**Authors:** Junbiao Dai, Jef D. Boeke, Zhouqing Luo, Shuangying Jiang, Yizhi Cai

**Affiliations:** 1 CAS Key Laboratory of Quantitative Engineering Biology, Guangdong Provincial Key Laboratory of Synthetic Genomics and Shenzhen Key Laboratory of Synthetic Genomics. Shenzhen Institute of Synthetic Biology, Shenzhen Institutes of Advanced Technology, Chinese Academy of Sciences, Shenzhen, 518055 China; 2 Institute for Systems Genetics, NYU Langone Health, New York, NY 10016 USA; 3 Manchester Institute of Biotechnology, University of Manchester, 131 Princess Street, Manchester, M1 7DN UK

Recent improvements in DNA synthesis and editing techniques enable engineering the entire genome of an organism, offering new tools to directly probe relationships between genotype and phenotype. Genome synthesis potentially allows the researchers to gain a much greater degree of control of an organism, and it also leads to a completely new way to understand the biology of genomes. In 2008, the first mega-size bacteria genome was built from oligonucleotides [1]. Next, the 4-Mb genome of *E. coli* was redesigned and engineered [2, 3]. More recently, the synthesis of first eukaryotic genome, the 12-Mb *Saccharomyces cerevisiae* genome, is nearing completion as the goal of the Sc2.0 initiative [4] and Genome Project-Write (GP-Write) has been proposed to engineer higher eukaryotes with gigabase-sized genomes [5].

As genome sizes increase, the design principles of synthetic genomes are becoming more sophisticated and complex. In the first synthetic genome, the *Mycoplasma* genome, only few watermarks were introduced [1]. The nearly completed Sc2.0 project involves building a genome that is heavily modified [4]. These modifications include the removal of all retrotransposons, subtelomeric repeats, and introns; eliminating and relocating all tRNA genes; swapping all TAG stop codon to TAA; and introducing numerous PCRTags (a type of watermark) by synonymous recoding of coding sequences. More importantly, over 4000 LoxPSym sites need to be inserted in the 3′ UTR of all non-essential genes, as well as at synthetic “landmarks,” a system designated as “synthetic chromosome rearrangement and modification by loxP-mediated evolution” (SCRaMbLE [6]). Overall, the native genome will be reduced in size by about 8% with an aim to reduce genomic contents and stabilize the genome, while still maintaining similar 3D structures and functions as wild-type chromosomes.

One thing we learned while constructing new chromosomes is that despite the variety of changes introduced, cells are quite tolerant to these perturbations. For example, the relocation of the megabase-size, highly repetitive ribosomal gene cluster on chrXII to a much smaller chromosome, chrIII, conferred only very minor, if any, effects on cell growth [7]. These results lead us to propose a new hypothesis that the yeast genome contains a larger variety of redundant elements. Therefore, more radical changes might be introduced to generate a much more compact genome. Here, we present a proposal to design and synthesize the next version of the synthetic yeast genome, dubbed Sc3.0.

## Sc3.0 genome design

Sc2.0 is designed based on the wild-type *S. cerevisiae* reference genome, but we propose that Sc3.0 relies on the prior completion of the Sc2.0 genome. As a first step, all essential genes from each chromosome will be restructured with designated regulatory elements. Next, each could be functionally validated and assembled into a dedicated chromosome with altered gene orders. These projects could be carried out by the Sc2.0 groups that originally synthesized them. Finally, the newly synthesized Sc3.0 chromosomes could then be combined into a single yeast to obtain strains with multiple chromosomes [8], or alternatively, these chromosomes could be merged into a single large chromosome [9, 10]. However, it is possible that a high density of synthetic lethal interactions would show up when such downsized chromosomes are combined [11]. We expect that multiple versions of the Sc3.0 base chromosomes could ultimately be generated, depending on which genes remain in each chromosome after SCRaMbLE. However, we propose here a single, more conservative Sc3.0 strategy that bypasses the challenges articulated above and can serve as a starting point for many subsequent variations. The core of the plan is the building and exploitation of the eArray, a circular centromere-containing DNA containing all of the essential genes, or a linear chromosome derived from it, synE, which we describe below.

## Constructing essential gene arrays (eArray)

The SCRaMbLE system allows for the stochastic generation of deletions, duplications, inversions, and translocations, among which deletions are desirable for the purpose of genome minimization. However, given the scattered distribution of essential genes throughout the genome, SCRaMbLE of haploid strains bearing one or more synthetic chromosomes often results in a high lethality rate by deleting one or more essential genes [6, 12]. To overcome this problem, we will first relocate all ~ 1000 essential genes, including their regulatory sequences from each chromosome, to a centromeric plasmid. These essential gene arrays (eArray) could be constructed by amplifying the desired sequences from the native genome by PCR and assembling them together using the yeast homologous recombination machinery; however, we propose synthesizing all the genes de novo, allowing for introduction of other systematic modification in DNA sequences, such as the use of promoters from sibling species which will contain variants to allow their distinction from the native promoters. This will allow for an orthogonal watermarking scheme, based on an alternative PCR-Tagging scheme that will allow three types of genes to be distinguished: native, Sc2.0, and Sc3.0 versions of each essential gene, which will be needed during construction. Very importantly, the eArray will be non-ScRaMbLEable, so it will remain intact through every future round of SCRaMbLE so that no essential genes will ever be lost. Subsequently, the function of each relocated gene in the eArray will be validated by tetrad analysis after transforming this plasmid into heterozygous diploid strains with one copy of the essential genes deleted.

It will be most efficient to synthesize the eArray directly in an Sc2.0 strain with all 16 Sc2 chromosomes. Once this is completed, the circular eArray can be converted to a linear chromosome containing all 1000 essential genes by use of the telomerator cassette [13]. This base strain represents a conservative place to start for 3.0 and can be SCRaMbLEd at will to explore genome minimization comprehensively.

## Genome minimization by SCRaMbLE

Previous studies using haploid strains directly for SCRaMbLE only identified clones with small regions removed, presumably due to the deleterious effects of losing essential genes [14]. The presence of eArray/synE could greatly enhance the variety of deletions, as we demonstrated recently (Luo et al., in submission). To further increase the power of deletion by SCRaMbLE, the *URA3* gene could be integrated at different locations throughout the synthetic chromosome in strains bearing eArray. After SCRaMbLEing, clones bearing at least one deletion including the *URA3* integration site were readily identified when they were selected in medium containing 5-FOA. We also identified deletions at other loci in these strains, suggesting it could be an efficient mechanism for chromosome minimization (Luo et al., in submission). Using this approach, the entire complement of the Sc2.0 chromosome could be minimized simultaneously in a new 3.0 version. After each round of SCRaMbLE, strains should be sequenced to identify regions remaining in the synthetic genome. Since SCRaMbLE is largely random, multiple rounds of SCRaMbLE will be needed. Sc3.0 thus represents the ultimate tool for driving to the most minimal of minimal *S. cerevisiae* genomes.

## Gene and chromosome reprograming—a multitude of possibilities

In order to generate a genome with few or no sequences from the native strain, after a chromosome is minimized after several rounds of SCRaMbLE, the remaining genes could be reprogrammed. We propose several principles to guide the Sc3.0 genome design. First, each open reading frame (ORF) could be recoded synonymously. The number of codons used in the designed genome could thereby be greatly reduced. Second, regulatory elements such as promoters and terminators should be replaced by functionally validated but completely artificial sequences, or sequences from other yeast species as proposed for the base Sc3.0 strain. Other intergenic sequences can then be removed, replaced by random sequences or corresponding sequences from other yeast species. We will gauge the quality of these sequences by carefully monitoring cell fitness. Non-coding RNAs (ncRNAs) and other known genetic elements can be replaced by orthologs from other yeast species. Intergenic sequences can be removed or replaced with random sequences of different lengths. The overall GC content can be retained—or not. Finally, genes can be clustered according to their functionality or arranged based on their chromosomal locations.

## Sc3.0 benefits and challenges

Through careful design, the Sc2.0 project has enabled experimental tests of many otherwise intractable questions about chromosome function and evolution. For example, removal of all retrotransposons and LTR repeats has produced a genome free of mobile elements, providing a system to assay effects of mobile elements on genome stability directly. Nevertheless, Sc2.0 made minimal changes to non-coding regions, no changes in gene order, and deletions of very limited number of genes. Complementary to Sc2.0, the Sc3.0 genome would allow to further explore questions such as how much of the yeast genome is redundant and could be compacted? What is the content of a minimal genome to support life under a given condition? Is the gene organization in the current genome evolutionary inevitable or contingent?

Given our current knowledge on the yeast genome, there remain many challenges. For example, engineering regulatory sequences is risky, since misregulation of any essential genes could lead to inviable cells. We expect much time will be spent on solving these issues. The available yeast genetics tools and directed evolution approaches will be extremely helpful. Additionally, making changes to most DNA sequences can cause long-range interactions to be disrupted, potentially resulting in dysfunction. In addition, many genes are co-regulated and it might be difficult to coordinate their expression using synthetic regulatory elements. We expect many of these challenges could be solved with experiences learned from the Sc2.0 project. We anticipate that all of the Sc2.0 chromosome synthesis teams will want to participate in this exciting next Sc3.0 phase.
